# Joint Analysis of Morphological Parameters and In Silico Haemodynamics of the Left Atrial Appendage for Thrombogenic Risk Assessment

**DOI:** 10.1155/2022/9125224

**Published:** 2022-03-14

**Authors:** Maria Isabel Pons, Jordi Mill, Alvaro Fernandez-Quilez, Andy L. Olivares, Etelvino Silva, Tom de Potter, Oscar Camara

**Affiliations:** ^1^Department of Information and Communication Technologies, Pompeu Fabra University, Barcelona 08018, Spain; ^2^Department of Radiology, Stavanger Medical Imaging Laboratory, Stavanger University Hospital, Stavanger 4036, Norway; ^3^Department of Quality and Health Technology, Faculty of Health Sciences, University of Stavanger, Stavanger 4036, Norway; ^4^Institute of Research and Innovation in Biomedical Sciences of the Province of Cadiz (INiBICA), Cadiz 11009, Spain; ^5^Department of Cardiology, Puerta Del Mar University Hospital, Cadiz 11009, Spain; ^6^Department of Cardiology, Cardiovascular Centre, Aalst 9300, Belgium

## Abstract

**Background:**

Atrial fibrillation (AF) is considered the most common human arrhythmia. In nonvalvular AF, around 99% of thrombi are formed in the left atrial appendage (LAA). Nevertheless, there is not a consensus in the community about the relevant factors to stratify the AF population according to thrombogenic risk.

**Objective:**

To demonstrate the need for combining left atrial morphological and haemodynamics indices to improve the thrombogenic risk assessment in nonvalvular AF patients.

**Methods:**

A cohort of 71 nonvalvular AF patients was analysed. Statistical analysis, regression models, and random forests were used to analyse the differences between morphological and haemodynamics parameters, extracted from computational simulations built on 3D rotational angiography images, between patients with and without transient ischemic attack (TIA) or cerebrovascular accident (CVA).

**Results:**

The analysis showed that models composed of both morphological and haemodynamic factors were better predictors of TIA/CVA compared with models based on either morphological or haemodynamic factors separately. Maximum ostium diameter, length of the centreline, blood flow velocity within the LAA, oscillatory shear index, and time average wall shear stress parameters were found to be key risk factors for TIA/CVA prediction. In addition, TIA/CVA patients presented more flow stagnation within the LAA.

**Conclusion:**

Thrombus formation in the LAA is the result of multiple factors. Analyses based only on morphological or haemodynamic parameters are not precise enough to predict such a phenomenon, as demonstrated in our results; a better patient stratification can be obtained by jointly analysing morphological and haemodynamic features.

## 1. Introduction

Atrial fibrillation (AF) is considered the most common of human arrhythmias. Approximately 2% of people younger than age 65 and about 9% of people aged 65 years or more have AF [1]. AF is currently seen as a marker of an increased risk of stroke since it favours thrombus formation inside the left atrium (LA). Around 99% of thrombi in nonvalvular AF are formed in the left atrial appendage (LAA) [2]. LAA shapes are complex and variable among the general population; researchers have sought to classify LAA morphologies and relate them to the risk of thrombus formation. The most established classification was initially proposed by Wang et al. [3] that classifies the LAA in four shapes: chicken-wing, cauliflower, windsock, and cactus. The authors reported that cauliflower and non-chicken-wing shapes were associated with more risk of thrombus formation [4]. Recently, another classification system [5] has been proposed based on the LAA angulation (LAA-H/L), defining as low-risk morphology (LAA-L) the ones with an acute angle bend or fold from the middle and proximal part of the LAA, and high-risk (LAA-H) otherwise. However, none of these classifications have achieved a scientific consensus due to their qualitative interpretation [6]. More quantitative anatomical factors based on the LAA orifice/ostium (interface between LA and LAA) such as its area, volume, minimum/maximum diameter, and irregularity, as well as LAA volume, width, and height, among others, have also been used for thrombogenic risk and stroke subtype stratification [7, 8].

At the same time, blood flow haemodynamics is a factor to consider for the assessment of the risk of thrombus formation, following Virchow's triad principles [9]. Low velocities and stagnated flow have been associated with the triggering of the inflammatory process and therefore the risk of thrombus generation [10, 11]. However, on daily clinical practice, LA haemodynamics can only be studied using echocardiography images, usually simplified to a single blood flow velocity value at one point in space and in time (e.g., centre of LAA ostium at end-diastole) [12]. Advanced imaging techniques such as 4D flow magnetic resonance imaging (MRI), allowing a more complete blood flow analysis, are emerging, but they still provide limited information in the left atria [11]. At this juncture, patient-specific models (e.g., digital twin concept [13]) based on computational fluid dynamics can provide a better haemodynamic characterization of the LA and LAA, deriving in silico indices describing blood flow at each point of the geometry over time. In the last decade, there have been several attempts to develop fluid simulation frameworks for the blood flow analysis of the human LA and LAA [14–16], but they have only been applied to a very limited number of patient-specific cases, except in a recent study with a cohort of simulated patients above fifty [17].

The aim of our study was to perform a joint analysis of morphological and in silico haemodynamic parameters from 30 patient-specific cases with and without history of transient ischemic attack (TIA) or cerebrovascular accident (CVA), to identify the combination of parameters that better predict TIA/CVA, as a potential surrogate of risk of thrombus formation and stroke.

## 2. Materials and Methods

### 2.1. Clinical Data

The initial patient cohort consisted of a total of 83 patients with AF from OLV Hospital in Aalst, Belgium. Nonvalvular patients (*n* = 71) were split into two groups based on whether or not they had suffered TIA/CVA; patients with valvular AF (*n* = 12) were discarded. All patients from our study underwent radiofrequency ablation of the pulmonary veins and were on permanent anticoagulation and gave written informed consent. The 3D LA models were acquired by reconstructing 3D rotational angiography images (3DRAs) obtained using an Innova 3D system (GE Healthcare, Chalfont St Giles, UK) and reconstructed into isotropic 3D volumes through the scanner workstation, providing isotropic 3D images with 0.23 mm or 0.45 mm volumetric pixel size for 512 or 256 pixels per dimension, respectively [18]. Segmentation of the left atria was achieved with semiautomatic thresholding and region-growing algorithms available at the scanner console.

**Figure 1:**
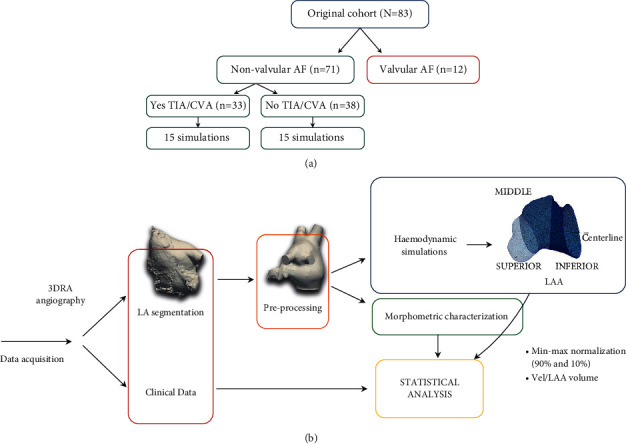
(a) Organization of the cohort of patients with atrial fibrillation (AF), transient ischemic attack (TIA), and cerebrovascular accident (CVA). (b) Computational pipeline followed in the study: data acquisition, left atrium (LA) segmentation from medical images, preprocessing, morphologic characterization of the LA and left atrial appendage (LAA), haemodynamic simulations, postprocessing, and statistical analysis of the data.

Morphological parameters were extracted from all nonvalvular patients. However, based on the quality of acquisitions, in silico simulations were only run on 30 patients (15 with history of TIA/CVA and 15 without), as shown in Figure 1(a). The available clinical data included weight, age, body mass index (BMI), body shape area (BSA), and the LAA morphology type (e.g., chicken-wing, cactus, windsock, or cauliflower, labelled by experts). The type of AF was also included, employing a distinction between paroxysmal or persistent (if lasting a maximum of 7 days or longer, respectively). The CHA2DS2-VASc score was assigned assuming that TIA/CVA cases had no history of thrombus before grading, which means that a high value of the score depends on other factors considered by the method. Mitral valve insufficiency was defined according to the angiographic grading and CHA2DS2-VASc score. A detailed breakdown of the steps followed in our study, including the advanced statistical classification, is shown in Figure 1(b).

### 2.2. Morphological Indices

**Figure 2:**
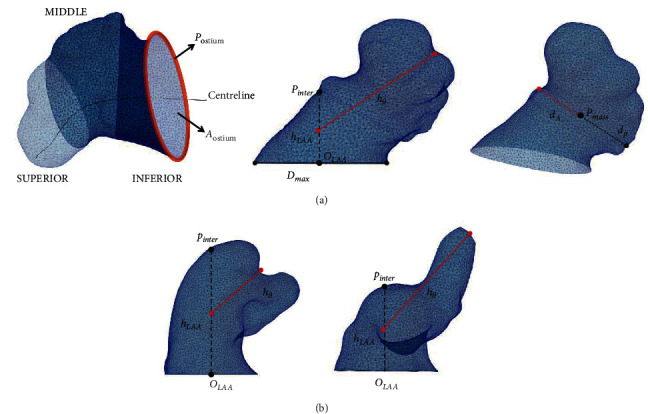
(a) Left atrial appendage division in three regions for the haemodynamic analysis (left) and morphological indices: ostium perimeter (p_ostium_) and area (A_ostium_), neck height (h_LAA_), distal point length (*h*_*θ*_), maximum ostium diameter (*D*_max_), origin of the LAA (O_LAA_), intersected point (p_inter_), anterior (*d*_A_) and posterior (*d*_P_) distance of the LAA and LAA, and centre of mass (p_mass_). (b) Examples of LAA with high and low tortuosity (left and right, respectively).

Before the automatic morphological characterization of the LAAs, a common reference system was defined for all LAAs by aligning the ostium plane with the *zx*-plane. The following were the analysed LAA morphological parameters (see Figure 2): the LAA volume and area; the neck height (*h*_*LAA*_), the distal point length (*h*_*θ*_), the LAA anterior and posterior distances (*d*_*A*_ and *d*_*P*_, respectively in Figure 2) between the LAA centre of mass (*p*_*mass*_) and the most distal points in the *x* direction, as well as its sum (anterior-posterior distance*, d*_*AP*_); the LAA centreline, using the VIDAA software [19]; and LAA tortuosity (*η*_*LAA*_). Additionally, the LAA ostium was characterized by its maximum and minimum diameters (*D*_max_ and *D*_min_, respectively) and its area and perimeter. Figure 2 shows a graphical representation of the estimated morphological parameters. Further details on the computation of morphological parameters are given in Supplementary Table S1 (see Figure S.1).

### 2.3. In Silico Haemodynamic Indices

Computational fluid dynamics (CFD) simulations with dynamic mesh movement of the mitral valve (MV) annulus ring were carried out by using ANSYS Fluent Solver 19.2 (Ansys, Inc., Pennsylvania, USA). In our study, we applied the boundary conditions (BCs) proposed by Mill et al. [20, 21]. They were defined as pressure inlet at the pulmonary veins (PVs) and as velocity outlet at the MV. A pressure waveform was extracted from one patient with AF in sinus rhythm through catheterization, while the velocity profile was extracted from a Doppler echocardiography acquisition; both conditions were applied to all simulated cases. Complete details on the 3D model construction and in silico simulation set-up are given in Supplementary Table S2 (see Figure S.2)

Haemodynamic indices from fluid simulations such as blood flow velocities were estimated averaging values of the second and third simulated beats (including systolic and diastolic phases) since the first one was used to stabilize the simulations. The time average wall shear stress (TAWSS), oscillatory shear index (OSI), relative residence time (RRT), and endothelial cell activation potential (ECAP; ratio between OSI and TAWSS (Equations (1)–(3)), with high values corresponding to low velocities and high flow complexity) were computed from the wall shear stress (WSS) at the LAA wall in order to identify the areas with high thrombogenic risk [22]. Finally, blood flow stagnation inside the LAA was assessed by integrating the flow rate at the ostium.

(1) $$\begin{array}{c} TAWSS=\;\frac{1}{T}\int_{0}^{T}\left|WSS\right|\text{d}t, \end{array}$$

(2) $$\begin{array}{c} OSI=\;\frac{1}{2}\frac{\int_{0}^{T}WSS\text{d}t}{\int_{0}^{T}\left|WSS\right|\text{d}t}, \end{array}$$

(3) $$\begin{array}{c} ECAP=\;\frac{OSI}{TAWSS}, \end{array}$$

(4) $$\begin{array}{c} RRT={\left[1-\left(2\cdot OSI\right)\cdot TAWSS\right]}^{-1}\;. \end{array}$$

Furthermore, a local analysis was also performed by dividing each LAA into three regions (see Figure 2): inferior (closest to the ostium), middle, and superior (farthest to the ostium). The LAA regional division was achieved by dividing the LAA centreline into three parts. The analysis of morphological and haemodynamic parameters was performed comparing control cases versus TIA/CVA, as well as chicken-wing versus non-chicken-wing LAA morphologies.

### 2.4. Statistical Analysis

The statistical analysis was performed using *R* Studio 1.2.1335 and can be divided into two main blocks: exploratory analysis and inferential analysis. Results are presented in terms of median (minimum–maximum) for continuous and non-normally distributed variables and mean ± standard deviation (SD) for continuous and normally distributed variables, whilst categorical data as characterized as count (percentage). Student's *t*-test, Mann-Whitney–Wilcoxon and Χ^2^ tests were used depending on the nature of the variable studied. The level of significance (*α*) was set to 0.05. Relevant parameters in terms of significant differences (*p* value <0.05) were considered potential risk factors. Afterwards, a stepwise regression model with only those morphological parameters that were statistically significant in the aforementioned statistical analysis was performed. For these stepwise regression models, the Akaike information criterion (AIC) [23] was used to study their prediction accuracy. A lower AIC represents a smaller information loss by the model, so the smaller the AIC, the higher the quality of the model. The output of the best morphology-based model was combined with the haemodynamic parameters to perform a joint analysis. Moreover, machine learning algorithms such as random forest were implemented to corroborate which parameters might play a critical role to classify cases in controls and TIA/CVA, as a complement to the classical statistical tests and regression models. The input for the random forest was the morphological parameters (LAA and LA volume, ratio LAAv/LAv, length of the centreline, LAA height, ostium anterior distance, maximum ostium diameter, ostium area, and LAA shape) found in the literature that contribute in the process of thrombus formation [4, 24–26], together with haemodynamic ones (ECAP, OSI, RRT, TAWSS, and velocity). Relevance parameters were obtained finding the best hyperparameters with a grid search procedure (number of trees = 500, variables sampled at each split = 4, maximum number of terminal nodes = 90). The Gini index was used to assess the importance of each predictor.

For the haemodynamic indices (ECAP, TAWSS, OSI, and RRT), the data were normalized applying a minimum–maximum sampling approach in all the studies. Percentiles 90% and 10% were discarded to be robust against outliers while analysing the whole LAA and its regions. For the blood flow velocity within the LAA, data were normalized by the total volume of the LAA in each case.

## 3. Results

### 3.1. Clinical and Morphological Analysis

No significant differences were found in AF persistent vs. paroxysmal cases (*p* = 1.00) between the TIA/CVA and control groups. On the same line, the prevalence of mitral insufficiency and the CHA2DS2-VASc score did not show significant differences (*p* = 0.832 and *p* = 0.343, respectively). The differences between both groups of patients were minimal, as can be seen in Table 1.

On the other hand, as detailed in Table 2, significant differences between TIA/CVA and control groups were found in morphological parameters such as the maximum (*p* < 0.001), minimum (*p* = 0.01), and mean (*p* = 0.001) diameters of the ostium, as well as its minimum radius (*p* = 0.02). The length of the LAA centreline was also significant (*p* = 0.05), as well as all ostium diameters, being larger in the TIA/CVA group. The LAA width, together with the anterior and anterior-posterior (AP) distances also showed significant levels (*p* = 0.01 and *p* = 0.02, respectively). The LAA shape was not significantly different between controls and TIA/CVA neither when classifying the LAA in chicken-wing versus non-chicken-wing (which were the majority of cases, 86%) nor in the classical four LAA types. Three TIA/CVA cases for the shape analysis were discarded due to the low quality of the reconstructed models and thus unclear shape. Morphological parameters obtained in the subset of cases where fluid simulations were run (see S3, Table S.3) showed the same trends, but fewer of them (maximum ostium diameters, ostium eccentricity, centreline length, and tortuosity) were statistically significant. The results of the regression model only including significant morphological parameters in the whole set of controls and TIA/CVA cases showed that the maximum ostium diameter, perimeter and area of the ostium, anterior distance, LAA area, and centreline length were the ones better differentiating TIA/CVA from control cases. However, the AIC obtained in morphology-based models was high among all the other ones (AIC = 85.96), thus on the lower range of accuracy.

### 3.2. Haemodynamic Analysis

**Figure 3:**
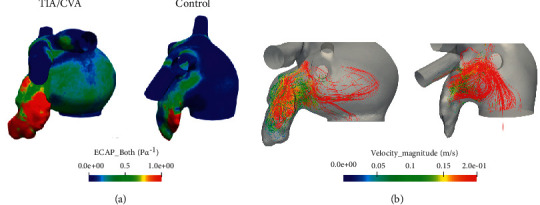
(a) Three-dimensional maps of endothelial cell activation potential (ECAP) for a transient ischemic attack or cerebrovascular accident patient (left) and a control case (right). High ECAP values (red areas) indicate a higher risk of thrombus formation due to low velocities and complex blood flow. (b) These velocities as well as the complexity of the flow within the LAA can be visualized with the streamlines for a thrombus case (left), while in a control case (right) flow remains in the ostium and does not reach the tip part of the LAA.

Figure 3 shows three-dimensional maps of ECAP of two exemplary TIA/CVA and control cases, in which the highest ECAP values (red areas in the figure) are located in the LAA regions in both cases. Lower blood flow velocities in the whole LAA were estimated in the TIA/CVA group compared with controls, as summarised in Table 3. In addition, higher ECAP, OSI, and RRT, all indicators of a higher risk of thrombus formation, were found in the TIA/CVA group. The TIA/CVA group also presented a worse flow washout from the LAA, indicating a higher percentage of stagnated blood in comparison with the control group (19.77% vs. 12.39%, respectively). Despite these trends, differences were not statistically significant between controls and TIA/CVA groups for any of the estimated haemodynamic indices. In all patients, vortex structures were visually present, but no substantial qualitative differences were found in terms of vorticity between TIA/CVA and control groups.

Analysing the LAA geometry per regions, blood flow velocities progressively decreased from the ostium to the superior part of the LAA (i.e., closer to the LAA tip) for both groups, still with higher values for controls. Conversely, haemodynamic indices were higher in the superior region for both groups, with larger differences between them.

Table 4 shows the in silico haemodynamic indices for each region of the LAA for chicken-wing and non-chicken-wing patients. Higher blood flow velocities, statistically significant (*p* = 0.04), were obtained for the chicken-wing group vs. the non-chicken-wing cases, consistently decreasing from the ostium to the superior LAA region. On the other hand, most of the remaining haemodynamic indicators of thrombogenic risk (ECAP, OSI, RRT) were higher in the chicken-wing group, led by large differences in the superior part of the LAA.

### 3.3. Joint Analysis of Morphological and Haemodynamic Parameters

**Figure 4:**
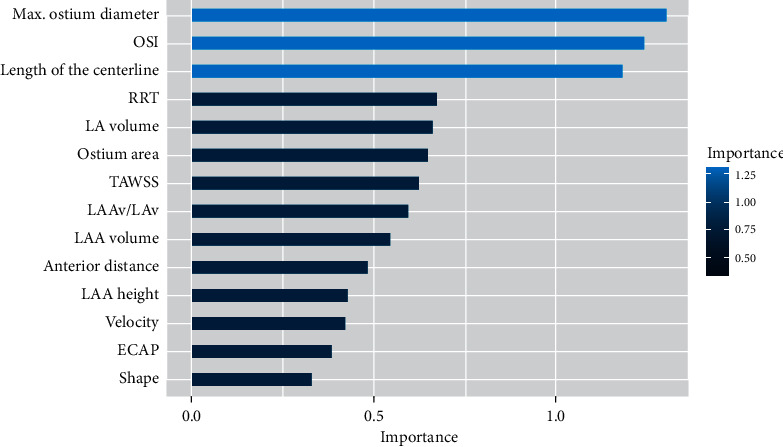
Model of the random forest performance in terms of Gini index. OSI: oscillatory shear index; RRT: relative residence time; LAAv: left atrial appendage volume; LAv: left atrium volume; TAWSS: time average wall shear stress; ECAP: endothelial cell activation potential.

The joint analysis reported that the most significant morphological indices (e.g., ostium characteristics, LAA area, and centreline length) were always good predictors of TIA/CVA. However, when in silico haemodynamic indices were added to the analysis, the results substantially improved the AIC metric obtained with morphological indices alone. A model with an AIC of 14 was obtained when adding haemodynamic indices (versus 85.96 with only morphological ones) such as RRT and TAWSS, along with some morphological indices (LAA volume, ostium area, anterior distance, and length of the centreline) that were reported as potential indicators of TIA/CVA history. The most distinguished features used for the region partition of each tree in the random forest were similar to those factors found in the previous statistical analysis. As can be seen in Figure 4, the maximum ostium diameter, OSI (i.e., flow complexity), and the length of the centreline were reported as good candidates to be predictors of thrombus formation among others. Analysing the model, the classification accuracy achieved was 70%.

## 4. Discussion

In this study, we assessed the significance of parameters characterizing the LAA morphology and haemodynamics to distinguish TIA/CVA from control cases. Factors representing Virchow's triad such as stasis, changes in the atrial geometry, specific LAA morphologies, and unfavourable haemostatic milieu are all likely to contribute to thrombus formation and thereby stroke risk [27].

Nevertheless, currently there is not any robust approach in AF patients to predict the risk of events potentially leading to stroke such as TIA/CVA or thrombus formation. Despite being regularly used, it has been proven that scores such as the CHA2DS2-VASc are not fully reliable, with some patients having low score values still generating thrombus [28]; the CHA2DS2-VASc resulted to be not significant in our study. Characterizing LA/LAA morphology with qualitative, subjective, or too simple parameters is also insufficient to capture the high complexity and large variability of LAA shapes. The classical LAA morphology type classification (e.g., chicken-wing, etc.) is not rigorous enough (i.e., high interobserver variability), leading to confronted results [6, 29, 30] when related to the risk of thrombus formation. In the studied database, we did not find differences in the percentage of chicken-wing LAA morphologies in controls and TIA/CVA groups, disagreeing with di Biase et al. [4] findings (e.g., less likelihood of an embolic event for chicken-wing LAA morphologies). Nevertheless, our models were extracted from angiography images that have a low resolution, and thus a precise assessment of the LAA shape might not be possible. In addition, when introducing the variable shape in our random forest, its importance was minimal.

On the other hand, other volumetric and morphological parameters such as ostium measurements (i.e., diameters, radius, area, perimeter), the LAA area/volume, and the centreline length were found to be good predictors of TIA/CVA events, in agreement with other studies [24, 25], suggesting a higher risk for larger LAA and ostium. However, Khurram et al. [29] found smaller LAA and ostium being more associated with lower thrombogenic risk. In our study, despite combining multiple clinical data and morphological parameters, the statistical studies produced regression models with limited accuracy, represented as high AIC metric values. As a direct consequence of this inaccuracy, the obtained parameters as potential risk factors might be unreliable.

The in silico haemodynamic indices resulting from our simulations showed trends in agreement with literature [4], i.e., TIA/CVA cases being associated with lower blood flow velocities, more complex patterns, larger residence times, and worse flow washout than controls. Nevertheless, all simulations were run with the same generic boundary conditions, which prevented more personalised outcomes that could be obtained with patient-specific boundary conditions (e.g., mitral valve velocity profile from Doppler studies). Additionally, differences were not statistically significant due to the large variability in each cohort (see standard deviations in Table 3) and the small number of cases where simulations could be run. In addition to the patient-specific boundary conditions, it could be interesting to study the effect of anticoagulation treatment on thromboembolic events in long term using in silico models.

When comparing chicken-wing vs. non-chicken-wing LAA morphologies, the latter presented lower blood flow velocities over the whole LAA, with statistical significance, in concordance to research assigning a protective role towards thrombus formation to chicken-wing LAA [4]. However, the regional analysis showed that the remaining haemodynamic indices were higher (i.e., more risk of thrombus formation) in the superior part of the LAA in chicken-wing morphologies, due to their particular elongated shape favouring complex flows and stagnation.

The joint analysis of morphological and haemodynamic indices achieved a better fitted predictive model than when analysed separately, with a substantial reduction of the AIC metric (from 85.96 with only morphological parameters to 14 when adding optimal haemodynamic indices). The model was obtained combining morphological features characterizing ostium and LAA size (e.g., the maximum ostium diameter, LAA area, and centreline length) together with haemodynamic indices of the whole LAA, mainly representing blood flow velocity magnitude (e.g., TAWSS and Vel/LAAv values). The results of the random forest algorithm reported that the maximum ostium diameter, OSI, and length of the centreline could be potential predictors of thrombogenic risk.

The present study demonstrates the benefit of using quantitative descriptors of blood flow patterns in the LA for the prediction of thrombogenic risk. However, obtaining in-vivo patient-specific data to fully characterize the 4D nature of LA/LAA haemodynamics is not yet possible in clinical routine. Computational simulations and digital twin models [13] offer an interesting alternative to derive in silico indices to be combined with morphological parameters from medical images for a personalised estimation of thrombogenic risk for a given patient. However, access to good-quality imaging data to build 3D models (e.g., geometry and boundary conditions) is not easy. Moreover, the whole fluid modelling pipeline is usually computationally demanding, requiring access to advanced hardware infrastructures and including tedious manual steps, preventing the processing of large amounts of cases. Nevertheless, we developed a modelling pipeline [19] to generate geometry-specific simulations in one working day.

This study has focused on studying the influence of LA/LAA morphology and in silico haemodynamics on thrombus formation before the implantation of a LAAO device, which can lead to a better patient selection and personalised therapy choice. However, the developed modelling pipeline to create haemodynamics simulations has also shown [17–21] to be useful in determining the formation of thrombus after the implantation of LAAO devices (i.e., device-related thrombus). Unfortunately, the required follow-up data to perform such verification were not available in the analysed patients in this study.

Some limitations of this study should be taken into consideration. First, the analysed cases were divided according to TIA/CVA history, which does not necessarily involve thrombus formation or degradation to stroke. In addition, we did not know if the TIA/CVA had its origin in the LAA. Moreover, the available imaging data were acquired with a 3DRA system, which has lower spatial resolution than computerized tomography (CT) scans and prevented the building of in silico simulations for all the available cases. The drawback of the 3DRA is that the obtained image quality highly depends on factors such as contrast injection. However, 3DRA with the use of contrast offers a reconstruction of the left atrial geometry in a more precise way (easily segmented with simple image processing tools) than 3D echocardiographic images, which is key to build computational models and run fluid simulations. Our study emphasises the significance of having patient-specific data as boundary conditions, e.g., from Doppler echocardiography, for having more realistic fluid simulations; as they were not available in the analysed cohort, some haemodynamic indices were not significantly different between TIA/CVA and control cases.

## 5. Conclusions

Thrombus formation in the LAA, potentially leading to transient ischemic attacks, cerebrovascular accidents, and stroke, results from the combination of different factors, including morphology and haemodynamics. However, their independent analysis does not offer the necessary holistic view to properly understand the underlying pathophysiological mechanisms and to estimate thrombogenic risk on an individual basis. We demonstrate in our study that the joint analysis of morphological parameters and in silico haemodynamic indices provides a better stratification of patients with and without TIA/CVA history. Relevant factors included the maximum ostium diameter and centreline length as well as in silico haemodynamic indices capturing blood flow complexity and magnitude values. The TIA/CVA group was associated with larger LAA and ostium as well as with lower blood flow velocities and more complex flow patterns, as assessed by various in silico haemodynamic indices. Furthermore, chicken-wing LAA morphologies presented higher blood velocities than non-chicken-wing ones.[31–34]

## Figures and Tables

**Table 1:** Clinical features of patients with and without a history of TIA/CVA

Characteristics	Control (*n* = 38)	TIA/CVA (*n* = 33)	*p* value
Age (years)	62.6 ± 8.9	67.2 ± 9.4	**0.035**
BMI (kg/m^2^)	28.4 ± 4.6	26.5 ± 4.8	0.097
BSA (m^2^)	2.0 ± 0.25	2.0 ± 0.25	0.589
Weight (kg)	86.4 ± 15.9	78.5 ± 16.6	**0.045**
*Gender*			
Female	11 (28.9%)	19 (57.6%)	**0.019**
Male	27 (71.1%)	14(42.4%)	

*AF type*			
Paroxysmal	25 (65.8%)	21.0 (63.6%)	1.000
Persistent	13 (34.2%)	12 (36.4%)	

*CHA2DS2-VASc* ^*∗*^			
0	9 (23.7%)	5 (15.2%)	0.343
1	9 (23.7%)	5 (15.2%)	
≥2	20 (52.6%)	23 (69.7%)	

*Mitral insufficiency*			
No	20 (52.6%)	15 (45.5%)	0.832
Grade 1	15 (39.5%)	14 (42.4%)	
Grade 2	3 (7.9%)	4 (12.1%)	

BMI = body mass index, BSA = body shape area, CVA = cerebrovascular accident, TIA = transient ischemic attack. Results are presented as mean ± SD or *n* (%). ^*∗*^Current TIA/CVA excluded.

**Table 2:** Volumetric and morphological features of patients with and without history of TIA/CVA.

Characteristics	Control (*n* = 38)	TIA/CVA (*n* = 33)		*p* value	
Max. ostium diameter (mm)	25.77 ± 4.40	30.20 ± 4.90		**<0.001**	
Min. ostium diameter (mm)	17.46 ± 3.66	19.63 ± 2.95		**0.01**	
Mean ostium diameter (mm)	21.62 ± 3.70	24.91 ± 3.66		**0.001**	
Min. ostium radius (mm)	6.99 ± 2.37	8.20 ± 1.81		**0.02**	
Ostium area (mm^2^)	367.18 ± 125.16	486.35 ± 148.42		**<0.001**	
Ostium perimeter (mm)	71.16 ± 12.27	80.99 ± 12.14		**0.001**	
Eccentricity	0.32 ± 0.10	0.34 ± 0.08		0.24	
LAA height (mm)	14.80 (9.18–22.93)	15.34 (8.83–30.63)		0.25	
Length of the centreline (mm)	34.48 ± 7.06	38.82 ± 8.74		**0.05**	
Tortuosity of the centreline	0.79 (0.50–0.96)	0.77 (0.45–0.88)		0.36	
LAA anterior distance (mm)	13.87 ± 3.75	16.03 ± 3.18		**0.01**	
LAA posterior distance (mm)	11.38 ± 3.17	12.83 ± 3.54		0.08	
LAA anterior-posterior distance (mm)	25.26 ± 6.70	28.86 ± 6.38		**0.02**	
Bending (degrees)	114 ± 17.55	103 ± 23.84		0.07	
Atrium volume (ml)	163 (82–256)	164 (100–269)		0.94	
LAA area (mm^2^)	2225 (1183–3918)	2677 (1747–6006)		**0.03**	
LAA volume (ml)	6.63 (2.59–15.50)	8.09 (4.12–15.88)		**0.02**	
LAA shape^*∗*^				
(i) Chicken-wing (ii) Non-chicken-wing	6 (15.79%) 32 (84.21%)	4 (13.33%) 26 (86.67%)		0.53 0.45	
*LAA shape* ^*∗*^				
(i) Chicken-wing	6 (15.79%)	4 (13.33%)		0.53	
(ii) Cauliflowers	10 (26.32%)	8 (26.67%)		0.82	
(iii) Cactus	13 (34.21%)	7 (23.33%)		0.26	
(iv) Windsock	9 (23.68%)	11 (36.67%)		0.82	

AP = anterior-posterior; CVA = cerebrovascular accident; Max = maximum; Min = minimum; TIA = transient ischemic attack. ^*∗*^Data were unclear for 3 TIA/CVA cases. Results were presented as mean ± SD or median (min–max) or *n* (%).

**Table 3:** Haemodynamic indices (mean ± standard deviation) for the whole LAA in controls and TIA/CVA groups.

	Control	TIA/CVA	*p* value
TAWSS (Pa)	0.31 ± 0.12	0.35 ± 0.14	0.40
ECAP (1/Pa)	0.87 ± 0.61	1.08 ± 0.69	0.35
OSI	0.15 ± 0.05	0.17 ± 0.04	0.17
RRT (s)	10.03 ± 5.97	11.38 ± 7.04	0.62
Vel/LAAv (m·ml/s/ml)	0.99 ± 0.80	0.84 ± 0.35	0.93

CVA = cerebrovascular accident; ECAP = endothelial cell activation potential; LAAv = left atrial appendage volume; OSI = oscillatory shear index; RRT = relative resident time; TAWSS = time average wall shear stress; TIA = transient ischemic attack; Vel = velocity. Higher values of ECAP, OSI, and RRT as well as lower values of TAWSS and Vel/LAAv indicate a higher risk of thrombus formation.

**Table 4:** Haemodynamic indices (mean ± standard deviation) for the whole LAA and each region for chicken-wing (CW) and non-chicken-wing (Non-CW) LAA shapes.

	Inferior		Middle		Superior		Whole	
	Non-CW	CW	Non-CW	CW	Non-CW	CW	Non-CW	CW
TAWSS (Pa)	0.51 ± 0.22	0.50 ± 0.14	0.26 ± 0.13	0.23 ± 0.09	0.11 ± 0.08	0.11 ± 0.10	0.33 ± 0.14	0.33 ± 0.07
ECAP (1/Pa)	0.48 ± 0.22	0.47 ± 0.20	1.11 ± 0.86	0.96 ± 0.47	4.30 ± 6.13	5.67 ± 5.12	0.92 ± 0.61	1.17 ± 0.80
OSI	0.16 ± 0.05	0.17 ± 0.06	0.14 ± 0.06	0.14 ± 0.06	0.12 ± 0.05	0.14 ± 0.04	0.16 ± 0.05	0.17 ± 0.06
RRT (s)	4.96 ± 2.02	4.82 ± 1.56	12.52 ± 8.52	11.22 ± 5.42	49.88 ± 61.59	68.51 ± 59.17	10.23 ± 6.32	12.25 ± 7.12
Vel/LAAv (m·ml/s)	**1.18** **±** **0.83**	**1.47** **±** **0.32**	**0.50** **±** **0.43**	**0.67** **±** **0.17**	**0.25** **±** **0.30**	**0.29** **±** **0.25**	**0.85** **±** **0.67**	**1.11** **±** **0.26**

CW = chicken-wing; ECAP = endothelial cell activation potential; LAAv = left atrial appendage volume; OSI = oscillatory shear index; RRT = relative resident time; TAWSS = time average wall shear stress; TIA = transient ischemic attack; Vel = velocity. Results with statistical differences are in bold. Higher values of ECAP, OSI and RRT as well as lower values of TAWSS and Vel/LAAv indicate a higher risk of thrombus formation.

## Data Availability

The computational fluid dynamic models, morphological database, haemodynamic database, and *R* studio script files data used to support the findings of this study are available from the corresponding author upon request.

## Abbreviations

AF: Atrial fibrillation
BCs: Boundary conditions
CFD: Computational fluid dynamics
CVA: Cerebrovascular accident
ECAP: Endothelial cell activation potential
LA: Left atrium
LAA: Left atrial appendage
MV: Mitral valve
OSI: Oscillatory shear stress
PVs: Pulmonary veins
RRT: Relative residence time
TAWSS: Time average wall shear stress
TIA: Transient ischemic attack.

## Supplementary Materials

Supplementary Materials In Supplementary Materials, a description of the computation of the morphological parameters studied, as well as examples of LAA morphologies studied (S1) is presented. In addition, the pipeline followed to perform the 3D model construction of the LA geometries and the set-up of the in silico simulations (S2); and finally, the results of the statistical analysis of all the volumetric and morphological parameters of the cases simulated (S3) .
