# Supplementary material for: Joint Analysis of Morphological Parameters and In Silico Haemodynamics of the Left Atrial Appendage for Thrombogenic Risk Assessment
Source: J Interv Cardiol. 2022 Mar 14;2022:9125224. doi: 10.1155/2022/9125224 (PMC8938090; doi:10.1155/2022/9125224)
Supplement: Supplementary Materials — In Supplementary Materials, a description of the computation of the morphological parameters studied, as well as examples of LAA morphologies studied (S1) is presented. In addition, the pipeline followed to perform the 3D model construction of the LA geometries and the set-up of the in silico simulations (S2); and finally, the results of the statistical analysis of all the volumetric and morphological parameters of the cases simulated (S3) . [file 9125224.f1.docx]

**SUPPLEMENTARY MATERIALS**

**S1: Morphological indices**

The ostium (orifice) of each LAA was characterized by maximum and minimum orifice diameter, minimum orifice radius, orifice area and orifice perimeter. The maximum diameter corresponds to the maximum Euclidean distance between the points from the contour of the ostium. Other parameters computed were the LAA area and volume, the neck height (*h_LAA_*), defined as the distance between the ostium centre (*O_LAA_*) and the intersected point (*p_inter_*), the latter obtained from a perpendicular line from the ostium plane (starting *at O_LAA_*), to the LAA wall touching point; the distal point length (*h_θ_*), which corresponds to the distance between the mean point of the *h_LAA_* and the most distal point of the LAA surface from the ostium (LAA apex); the LAA anterior and posterior distances (*d_A_* and *d_P_*, respectively), as well as its sum (anterior-posterior distance, *d_AP_*); the LAA centreline length (from the ostium to the LAA apex); and LAA tortuosity (*η_LAA_*), which is the ratio between the neck height (*h_LAA_*) and the sum of the distal point length and half of the neck height:

$$\hat{\eta_{LAA}}=\frac{\hat{h_{LAA}}}{\frac{\hat{h_{LAA}}}{2}+\hat{h_{\theta}}}$$

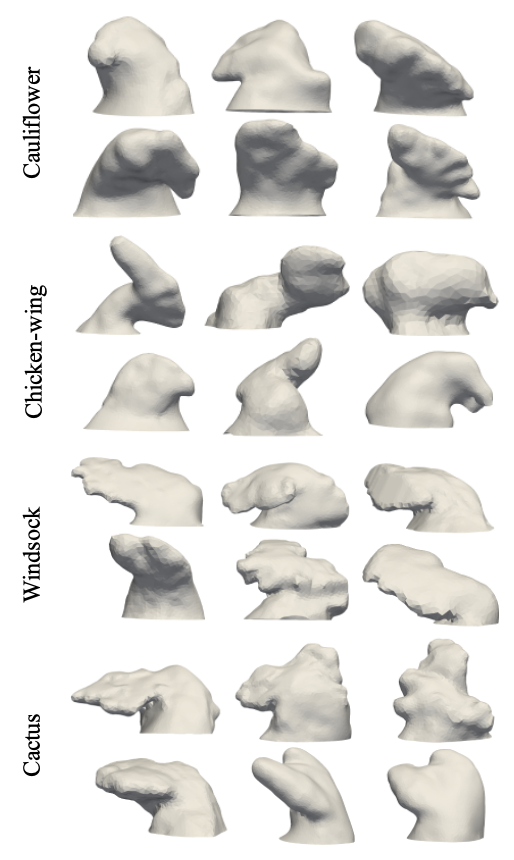


**Figure S.1.** Representation of the LAA classification based on their shape for 6 different cases in each group: cauliflower, chicken-wing, windsock, and cactus.

**S2: 3D model construction and in-silico simulations set-up**

The left atrium models were segmented from 3D rotational angiography (3DRA) images. Due to the low quality of the reconstructed atrium, the original LA segmentations had irregularities. Therefore, the geometries were smoothed using the Taubin filter smoothing algorithm surface (*λ* = 0.6, *μ* = –0.53)^32^ and adding final manual corrections if required. The softwares used to reconstruct the models were Meshlab 2016.12, and Meshmixer 3.5. Since the pulmonary veins (PV) could not be segmented properly from 3DRA data due to the lack of contrast inside the veins at the moment of acquisition, the PVs were reconstructed adding tubes but respecting the original orientation.

CFD simulations were carried out with ANSYS Fluent Solver 19.2 (Ansys, Inc. Pennsylvania, USA). Blood was modelled as an incompressible Newtonian fluid with density 1060 kg/m^3^ and viscosity 0.0035 Pa/s.A laminar model was used since the Reynolds numbers, which were computed at the MV and PV, were within a laminar regime. The flow could be considered as Newtonian since the shear rate had values higher than 100s^-1 33^. The time step was set to 0.01s. Residuals for continuity equations were set as 0.005 for convergence criteria. The dynamic mesh method was the spring-based method available in Fluent based on a MV ring function described in the work of Veronesi et al^34^. Figure B.1 represents the boundary conditions applied: pressure-inlet at the pulmonary veins, and velocity-outlet at the mitral valve.


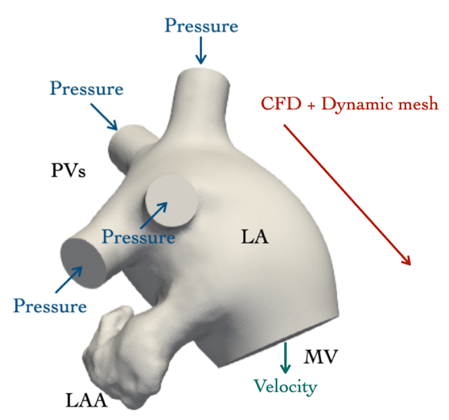


**Figure S.2** Boundary conditions at the left atrium (LA) and left atrial appendage (LAA): Computational Fluid Dynamics (CFD) geometrical model with velocity outlet at the mitral valve (MV) and pressure inlet at the pulmonary veins (PVs).

**S3: Global statistical analysis**

**Table S.3** Volumetric and morphological features of simulated patients with and without history of TIA/CVA.

| Characteristics | Control (n=15) | TIA/CVA (n=15) | p-value |
| --- | --- | --- | --- |
| Max. ostium diameter (mm) | 26.32 ± 5.26 | 31.18 ± 5.12 | **0.02** |
| Min. ostium diameter (mm) | 18.76 ± 4.25 | 19.59 ± 3.18 | 0.55 |
| Mean ostium diameter (mm) | 22.54 ± 4.53 | 25.38 ± 3.94 | 0.10 |
| Min. ostium radius (mm) | 7.77 ± 2.44 | 8.56 ± 1.84 | 0.33 |
| Ostium area (mm^2^) | 407.10 ± 154.56 | 518.72 ± 169.04 | 0.07 |
| Ostium perimeter (mm) | 73.62 ± 14.30 | 83.24 ± 13.41 | 0.07 |
| Eccentricity | 0.28 ± 0.10 | 0.37 ± 0.06 | **0.02** |
| LAA height (mm) | 14.67 (11.52 – 22.93) | 16.07 (9.61 – 30.63) | 0.77 |
| Length of the centreline (mm) | 34.48 ± 6.99 | 42.40 ± 9.38 | **0.02** |
| Tortuosity of the centreline | 0.81 (0.57 – 0.96) | 0.72 (0.45 – 0.88) | **0.02** |
| LAA anterior distance (mm) | 14.85 ± 4.06 | 16.66 ± 2.81 | 0.17 |
| LAA posterior distance (mm) | 11.88 ± 3.64 | 12.97 ± 3.03 | 0.39 |
| LAA anterior-posterior distance (mm) | 26.78 ± 7.41 | 29.63 ± 5.44 | 0.24 |
| Bending (degrees) | 113.49± 14.43 | 99.78 ± 22.64 | 0.06 |
| Atrium volume (ml) | 168 (131 – 256) | 165 (115 -269) | 0.55 |
| LAA area (mm^2^) | 2583 (1215 – 3918) | 3329 (1747 – 6005) | 0.14 |
| LAA volume (ml) | 7.80 (2.85 – 15.50) | 10.40 (4.44 – 15.88) | 0.51 |
| LAA shape |  |  |  |
| - Chicken-wing - Non-chicken-wing | 3 (20 %)  12 (80%) | 4 (26.67%)  11 (73.33%) | 1.00  1.00 |
| LAA shape |  |  |  |
| - Chicken-wing | 3 (20%) | 4 (26.67%) | 0.74 |
| - Cauliflowers | 3 (20%) | 3 (20%) | 1.00 |
| - Cactus | 4 (26.67%) | 3 (20%) | 1.00 |
| - Windsock | 5 (33.33%) | 5 (33.33%) | 1.00 |

AP = anterior-posterior; Max = maximum; Min = minimum;

Results presented as mean ± SD or median (min-max) or n (%)
